# A Textual Mapping Analysis of Mass Trampling Incident Emergency Drill Scripts Based on ICN Core Competencies in Disaster Nursing Version 2.0

**DOI:** 10.1155/jonm/5309874

**Published:** 2026-10-29

**Authors:** Hanxi Chen, Guandie Hong, Xin Ma, Siyi Huang, Siyin Chen

**Affiliations:** ^1^ Department of Nursing Guangzhou Red Cross Hospital (Guangzhou Red Cross Hospital of Jinan University) Guangzhou China gzrch.com; ^2^ School of Nursing Guangdong Pharmaceutical University Guangzhou China gdpu.edu.cn; ^3^ Department of Public Health and Medicinal Administration Faculty of Medicine Unit of Psychiatry University of Macau Macao SAR China umac.mo

**Keywords:** core competencies, disasters, nursing, textual mapping

## Abstract

**Aim:**

To evaluate the alignment of mass trampling incident emergency drill scripts with the International Council of Nurses (ICN) Core Competencies in Disaster Nursing Version 2.0 and to identify areas of higher and lower textual coverage of competency items.

**Background:**

Crowd crush incidents are increasingly frequent worldwide and pose severe risks of mass casualties. Despite the importance of systematic and standardized emergency drills, there is limited evidence on the extent to which existing drill scripts address core disaster nursing competencies, particularly in high‐risk mass gathering scenarios.

**Methods:**

We mapped 24 provincial‐level emergency drill scripts from a mass casualty exercise in China against the ICN Version 2.0 framework. Two researchers independently reviewed each script line by line and deductively coded the content against the eight domains and 33 competency items of the ICN Version 2.0 framework. Coverage rates were calculated and summarized using four descriptive categories: high, moderate, low, and no textual coverage.

**Results:**

Domain‐level coverage represents the proportion of competency items appearing in at least one script. It was high for Assessment (100%), Incident Management (100%), Preparation and Planning (80%), and Communication (75%). Law and Ethics (67%), Intervention (50%), and Safety and Security (40%) met the criterion for moderate coverage. No domain fell within the low‐coverage category, and no Recovery‐domain item was represented in any script (0% domain‐level coverage). Several competency items related to post‐drill evaluation, education and training, psychological support, isolation, decontamination, and recovery were infrequently represented or absent.

**Conclusions:**

The drill scripts showed relatively strong textual representation in Assessment, Incident Management, Preparation and Planning, and Communication, but less comprehensive representation in Law and Ethics, Intervention, and Safety and Security, while Recovery was not represented. Future drills should strengthen underrepresented competency‐related content that can reasonably be demonstrated during immediate response. Competencies involving organizational planning, education and training, post‐exercise activities, and recovery‐phase activities may require complementary assessment beyond script review to support a more objective and comprehensive evaluation.

**Implications for Nursing Management:**

This study may help nursing managers understand how disaster nursing core competencies are represented in emergency drill scripts and identify competency areas that warrant further attention. The findings may provide a reference for targeted improvements in disaster preparedness planning, training, drill design, and subsequent competency assessment.

## 1. Introduction

Crowd crush injury, defined as physical compression or trampling injuries resulting from localized overcrowding due to collective movement in high‐density environments triggered by real or perceived threats [1], represents a severe disaster capable of causing mass casualties within short time frames and has global implications [2]. In recent years, such incidents have shown an upward trend, coinciding with the increase in large‐scale global events, including sports competitions, concerts, and public celebrations. Consequently, crowd crush has become a critical public safety issue [3]. For example, the 2021 Astroworld Festival crowd crush in Houston, USA, resulted in 10 fatalities and approximately 300 injuries [4]; the 2022 Halloween crowd crush in Itaewon, Seoul, South Korea, caused 159 deaths [5]; and the 2014 New Year’s Eve crowd crush at Chenyi Square on the Bund in Shanghai, China, led to 36 deaths and 49 injuries [6]. These events have not only caused significant casualties but also exposed deficiencies in the emergency response capacity, resource allocation, and interagency coordination of medical rescue efforts, highlighting the urgent need for systematic and standardized drills.

Emergency drills are activities conducted during the preparedness phase that simulate emergency scenarios based on relevant response plans [7]. In the healthcare field, drills not only test emergency systems and resource coordination but also provide a basis for improving plans and enhancing response capabilities [8, 9]. China’s “14th Five‐Year Plan for Emergency Medical Rescue” explicitly identifies the construction of medical emergency training bases as a core task to ensure rapid response [10]. In this context, rescue personnel can practice triage, on‐site care, and decision‐making processes in high‐pressure environments that closely mimic real incidents, thereby strengthening teamwork and disaster response capabilities [11, 12]. Multiple studies have evaluated drill effectiveness internationally; for instance, a full‐scale trauma simulation in Nepal assessed emergency preparedness and actual response capacity [13]. Bajow et al. [14] found that functional simulation exercises in a hospital in Riyadh helped identify systemic issues, clarify roles of medical staff, and improve team disaster response competence. Some medical institutions in China have also conducted similar drills, such as role‐playing exercises in Luozha County, Tibet, which enhanced nursing skills, emergency response ability, and nurse–patient communication [15]. However, most existing drills focus on operational skill training, with limited reporting on post‐drill evaluation and reflection, and a systematic drill framework specific to crowd crush incidents remains relatively underdeveloped.

To enhance nurses’ comprehensive competencies in high‐risk disaster events, the International Council of Nurses (ICN) and the World Health Organization (WHO) issued the CORE COMPETENCIES IN DISASTER NURSING VERSION 1.0 in 2009, which proposed that nurses should possess competencies in mitigation/prevention, preparedness, response, and recovery/rehabilitation (ICN, 2009) [16]. As the discipline of disaster nursing developed, ICN recognized that the initial framework was insufficient to reflect the skills required for advanced practice nurses. Consequently, in 2019, ICN released CORE COMPETENCIES IN DISASTER NURSING VERSION 2.0, updating and expanding Version 1.0. Competencies are categorized into Level I (for all registered nurses) and Level II (for advanced practice/specialist nurses), covering eight domains: preparation and planning, communication, incident management, safety and security, assessment, intervention, recovery, and law and ethics. This framework provides systematic theoretical and practical guidance for nurse training, emergency drill design, and competency evaluation [17]. Currently, the ICN 2.0 framework has been widely applied worldwide. For example, in Austria, Germany, and Switzerland, it underwent cultural adaptation and translation through multistage consensus processes to train nurses in disaster preparedness and planning competencies [18]. In China, advanced practice nurses employed the ICN 2.0 framework for competency assessment and training during the COVID‐19 pandemic, effectively enhancing comprehensive response capabilities [19]. Moreover, the framework informs educational curriculum design by providing standardized teaching and evaluation indicators for training institutions, ensuring that drills cover core competencies and enabling measurable and sustainable competency development [19, 20]. Collectively, these applications demonstrate the substantial theoretical and practical value of the ICN 2.0 framework globally.

Mapping is a systematic process of aligning literature or content with a predefined framework to evaluate coverage [21]. Text analysis involves processing unstructured text to identify themes and patterns. Text mapping analysis combines the two, enabling the assessment of competency coverage by aligning unstructured content with the framework [22]. Existing studies have applied text mapping to curricula, training manuals, and drill scripts to identify gaps in core competency coverage and guide improvements [20, 21]. For instance, Alanazi [11] developed a disaster drill evaluation questionnaire through content analysis to identify coverage of core competencies and inform drill design optimization. Motsepe et al. [21] assessed the content of emergency nurse training manuals to support training enhancements. Studies have also shown that combining the ICN Version 2.0 framework with text mapping analysis can evaluate the coverage of core competencies in curricula. Loke et al. [20] mapped a disaster nursing postgraduate curriculum, finding coverage rates of 83% for Level I and 69% for Level II competencies, verifying the framework’s applicability. However, although text mapping analysis has been widely used in curricula and training, its application to crowd crush emergency drill scripts remains underexplored.

Given China’s expanding economy and the increasing frequency of large‐scale events, strengthening safety management and medical rescue capacity in crowded settings is of considerable importance. Previous studies have applied the ICN Core Competencies in Disaster Nursing Version 2.0 to nursing work during disaster response in China. For example, Mao et al. [19] examined the representation of ICN disaster nursing core competencies in the actual response actions of three rescue teams during the COVID‐19 outbreak. In contrast, the present study evaluated written emergency drill scripts developed by 24 teams for the same standardized crowd crush mass‐casualty scenario. Written emergency drill scripts can be repeatedly reviewed and coded using the same ICN competency framework, enabling systematic comparison of the representation of disaster nursing core competencies across teams and allowing a more objective, standardized, and reproducible assessment. This approach can effectively avoid errors caused by a lack of homogeneity among evaluators during on‐site assessment and can also effectively avoid failure to capture the core competencies presented on site due to limitations in evaluators’ attention, energy, or field of view. Accordingly, based on the ICN Core Competencies in Disaster Nursing Version 2.0, this study aimed to evaluate the textual representation of disaster nursing core competencies in emergency drill scripts for crowd crush incidents and to provide evidence to inform nursing leadership, disaster preparedness planning, workforce development, competency assessment, and organizational decision‐making.

## 2. Methods

### 2.1. Study Design and Purpose

This descriptive textual analysis aimed to evaluate the alignment between scenario scripts from a provincial “Mass Casualty Exercise for Crowd Trampling Incidents” drill presentation and the “ICN CORE COMPETENCIES IN DISASTER NURSING VERSION 2.0(ICN CCDN V2.0)”. The drill presentation was jointly organized by the Guangdong Nurses Association and the Disaster Nursing Research and Development Committee of the Guangdong Lingnan Nightingale Nursing Research Institute on July 16, 2025. The primary objective was to map the exercise scripts of 24 participating teams to specific domains and competency items within the ICN CCDN V2.0 framework, thereby describing areas of higher and lower textual coverage of competency items in the written drill scripts.

### 2.2. Data Source and Collection

#### 2.2.1. Source

Formal authorization was obtained from both the Guangdong Nurses Association and the Disaster Nursing Research and Development Committee of the Guangdong Lingnan Nightingale Nursing Research Institute. Following this, all 24 drill presentation scenario scripts for the trampling incident emergency exercise were retrieved from the Committee’s archives. The drill presentation scenario was provided by the Disaster Nursing Committee of the Guangdong Nurses Association. Based on the assigned scenario and the Core Competencies in Disaster Nursing Version 2.0, each participating team independently developed a scenario script for a mass‐casualty stampede emergency drill. No cash prizes or other financial rewards were offered. All participating teams conducted and presented their drills based on the same scenario, and their performances were evaluated by a panel of experts. Based on the expert evaluations, first‐, second‐, and third‐prize awards were presented to outstanding teams in recognition of their drill presentations. The primary purpose of adopting this presentation format was to provide participating teams with a platform to conduct emergency drills, demonstrate their practices, exchange experiences, and learn from one another around a common disaster scenario. It also enabled assessment of their disaster emergency response capabilities and provided disaster emergency response professionals in Guangdong Province with opportunities for observation and learning. The participating medical teams exclusively comprised core units from: Grade A tertiary general hospitals within the province, National Emergency Medical Rescue Teams, Guangdong‐Hong Kong‐Macao joint response units, collectively representing the highest tier of medical response capabilities in the region.

#### 2.2.2. Inclusion Criteria

All complete scenario scripts submitted for this drill presentation cycle were included in the analysis.

#### 2.2.3. Ethical Processing

Scripts were anonymized by removing team names and institutional identifiers. Each script was assigned a unique identification code for analysis and reporting. Original files were stored on password‐protected electronic devices accessible only to research team members. The study protocol was approved by the Ethics Committee of Guangzhou Red Cross Hospital (Approval No.: KY2024‐607‐01).

### 2.3. Data Extraction and Analysis Process

This study adopted a structured deductive content analysis approach, which was implemented through the following steps:

1. Analytical framework: The research team reviewed the ICN Core Competencies in Disaster Nursing Version 2.0 (ICN 2.0) and specified its 8 domains and 33 competency items as the analytical framework. The complete set of competency items, together with their item numbers and domain assignments, is presented in Table S1. Readers may also refer to the original ICN 2.0 handbook (ICN, 2019) for the full framework. All 33 Level II competencies were retained to provide a comprehensive and standardized framework for mapping the drill scripts. However, not all competencies were expected to be equally demonstrable within the simulated scenario. Competencies requiring organizational planning, education and training, or post‐event activities were distinguished from those that could reasonably be demonstrated during the immediate response (Table S2).
2. Independent coding and framework mapping: Two researchers independently reviewed all 24 emergency drill scripts line by line using a standardized coding protocol based on the ICN 2.0 framework. ① First, they identified response measures related to trampling incident management described in each script; ② Second, they mapped the identified measures to the corresponding domains and items of the ICN 2.0 framework; ③ For competency items supported by relevant script content, they marked the status as “Covered” and recorded representative script excerpts and notes on underrepresented content where applicable; ④ Finally, they compiled a standardized dataset linking “script content,” “competency items,” “coverage status,” and “coding notes” for each entry. This process supported systematic and consistent data extraction.
3. Independent verification and inter‐rater agreement: Two researchers independently re‐evaluated the mapping of all 33 ICN Level II competency items across the 24 drill scripts using predefined coding criteria. A competency item was coded as “represented” only when the script contained explicit textual evidence sufficient to reflect the substantive requirement of that competency; the presence of a related emergency‐response activity alone was not considered sufficient if it did not meet the full requirement of the competency. Each script–competency pair was coded dichotomously as represented (1) or not represented (0). Inter‐rater agreement between the two researchers was assessed using percent agreement and Cohen’s kappa. After completing the independent coding, the two researchers compared their results and discussed any discrepancies with reference to the original script text and the full wording of the corresponding ICN competency until consensus was reached. Any disagreements that remained unresolved after discussion were adjudicated by a third researcher with expertise in disaster nursing. The first two researchers were postgraduate nursing students engaged in disaster nursing research, while the third researcher was a professor with extensive experience in disaster nursing research and practice. The final consensus coding was used to recalculate item‐level script coverage and domain‐level competency‐item coverage.
4. Quantification and Descriptive Classification: ① Coverage rate calculation: Item‐level coverage was calculated as the number of scripts in which a given ICN competency item was represented divided by the total number of analyzed scripts (*n* = 24). Domain‐level coverage was calculated as the number of competency items represented in at least one script within a given ICN domain divided by the total number of competency items in that domain. Therefore, item‐level coverage reflects the frequency with which each competency item appeared across the 24 scripts, whereas domain‐level coverage reflects whether the items within each ICN domain were represented in the analyzed script set. ② Descriptive classification of coverage levels: For descriptive presentation, coverage levels were classified into four categories adapted from a previous percentage‐based content analysis: high textual coverage (70%–100%), moderate textual coverage (40%–69%), low textual coverage (1%–39%), and no textual coverage (0%) [23]. The previous study used these percentage thresholds to describe the extent to which predefined practices and skills were represented across analyzed curriculum units. In the present study, these thresholds were adopted as descriptive categories to facilitate comparison across ICN competency domains and items. They should not be interpreted as validated psychometric cut‐off points or as criteria for judging actual nursing competence, institutional preparedness, or drill performance. Therefore, the terms used in this study refer only to the degree of textual representation of ICN competency items in the analyzed scripts. ③ Descriptive synthesis of script content: After calculating the coverage rates, recurring script components and underrepresented competency items were summarized descriptively. This synthesis focused on patterns of textual representation within the drill scripts and did not assess the actual implementation quality of the drills or the real‐world performance of participating nurses.

## 3. Results

Using the ICN Core Competency Framework as the benchmark, we mapped 24 emergency drill scripts from the “Mass Casualty Exercise for Crowd Trampling Incidents” to the eight core competency domains and 33 specific items. Item‐level coverage counts, noncoverage counts, and coverage rates were summarized accordingly. For descriptive presentation, the results were summarized using four descriptive categories: high textual coverage, moderate textual coverage, low textual coverage, and no textual coverage. These categories were used to describe the degree of textual representation of ICN competency items in the analyzed scripts.

### 3.1. Overview of Overall Coverage

Inter‐rater agreement between the two independent researchers was 98.0%, with a Cohen’s kappa of 0.956 (*p* < 0.001). Textual coverage varied across the eight ICN core competency domains and individual competency items (Table 1/Figure 1). Domain‐level coverage represents the proportion of competency items appearing in at least one script. For descriptive presentation, domain‐level textual coverage was summarized using four descriptive categories: high textual coverage (70%–100%), moderate textual coverage (40%–69%), low textual coverage (1%–39%), and no textual coverage (0%). To illustrate the textual mapping process, Table S3 presents an example from the Assessment domain. Table S4 illustrates the mapping of selected content from an emergency drill script to the corresponding ICN competency items, while Table S5 presents the original script. These categories were used only to describe the degree of textual representation of ICN competency items in the analyzed scripts and do not imply validated performance levels. Coverage also varied substantially among individual competency items within each domain. These categories were used only to describe the degree of textual representation of ICN competency items in the analyzed scripts and do not imply validated performance levels. Coverage also varied substantially among individual competency items within each domain.

**TABLE 1:** Domain‐level competency‐item coverage across ICN core competency domains.

ICN core competency domains	Competency items represented in ≥ 1 script, n	Total number of items(*n*)	Domain‐level competency‐item coverage (%)
Preparation and Planning	4	5	80
Communication	3	4	75
Incident Management	4	4	100
Safety and Security	2	5	40
Assessment	4	4	100
Intervention	3	6	50
Recovery	0	2	0
Law and Ethics	2	3	67

*Note:* Domain‐level coverage represents the proportion of competency items appearing in at least one script. These percentages do not represent the proportion of scripts demonstrating the domain. For descriptive interpretation, domain‐level competency‐item coverage was classified as high (70%–100%), moderate (40%–69%), low (1%–39%), or no coverage (0%), adapted from previous content‐mapping research [23].

**FIGURE 1:**
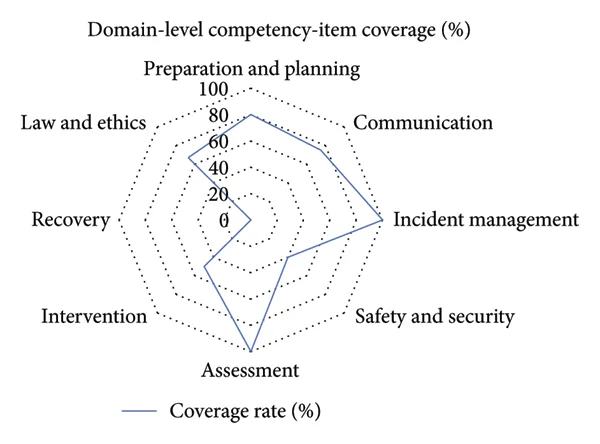
Radar chart of domain‐level competency‐item coverage across ICN core competency domains. Note. Percentages represent the proportion of competency items within each ICN domain that were represented in at least one of the 24 scripts, rather than the proportion of scripts demonstrating that domain.

#### 3.1.1. Domains With High Textual Coverage (70%–100%)

Among the eight ICN core competency domains, four domains had high textual coverage: Assessment, Incident Management, Preparation and Planning, and Communication. Assessment had 100% domain‐level textual coverage, with all four competency items represented in at least one script. Items 19 and 20 were covered in all 24 scripts, Item 22 was covered in 13 scripts, and Item 21 was covered in one script. Incident Management likewise had 100% domain‐level textual coverage, with all four competency items represented in at least one script. Item 13 was covered in 21 scripts, whereas Items 10, 11, and 12 were covered in six, two, and one scripts, respectively. Domain‐level textual coverage for Preparation and Planning was 80%, with four of five competency items represented in at least one script. Items 1 and 3 were covered in all 24 scripts, Item 4 was covered in 15 scripts, Item 2 was covered in one script, and Item 5 was not covered in any script. Communication reached 75% domain‐level textual coverage, with three of four competency items represented in at least one script. Item 6 was covered in 20 scripts, Item 7 was not covered, and Items 8 and 9 were each covered in two scripts.

#### 3.1.2. Domains With Moderate Textual Coverage (40%–69%)

Three domains met the criterion for moderate textual coverage: Law and Ethics, Intervention, and Safety and Security. Law and Ethics had 67% domain‐level textual coverage, with two of three competency items represented in at least one script. Items 32 and 33 were covered in all 24 scripts, whereas Item 31 was not covered in any script. Intervention had 50% domain‐level textual coverage, with three of six competency items represented in at least one script. Items 23 and 27 were each covered in 16 scripts, Item 28 was covered in 10 scripts, and Items 24, 25, and 26 were not covered in any script. Safety and Security had 40% domain‐level textual coverage, with two of five competency items represented in at least one script. Item 18 was covered in 22 scripts, Item 16 was covered in one script, and Items 14, 15, and 17 were not covered in any script.

#### 3.1.3. Domains With Low Textual Coverage (1%–39%)

No ICN competency domain fell within the low textual coverage category.

#### 3.1.4. Domains With No Textual Coverage (0%)

Recovery had 0% domain‐level textual coverage, with neither of its two competency items represented in any of the 24 scripts.

#### 3.1.5. Mapping Results by ICN Competency Domain (Table 2)

1. Preparation and Planning: Textual Coverage of Planning, Role Communication, Vulnerable Populations, Improvement, and Education‐Related Items

**TABLE 2:** Detailed coverage of the 33 items under ICN core competency domains.

ICN core competency domains	Core competencies items	Coverage details
Preparation and planning	1. Participates with other disciplines in planning emergency drills/exercises at the institution or community level at least annually	Coverage: 24 groups in total
	2. Plans nursing improvement actions based on results of drill/exercise evaluation	Coverage: 1 group in total (Group 20); Noncoverage: 23 groups in total
	3. Communicates roles and responsibilities of nurses to others involved in planning, preparation, response and recovery	Coverage: 24 groups in total
	4. Includes actions relevant to needs of vulnerable populations in emergency plans	Coverage: 15 groups in total; Noncoverage: 9 groups in total (Groups 2, 3, 4, 5, 10, 13, 14, 15, 16)
	5. Incorporates Level I core competencies in Disaster Nursing in any basic nursing education program or refresher course	Coverage: 0 groups in total

Communication	6. Plans for adaptable emergency/disaster communications systems	Coverage: 20 groups in total; Noncoverage: 4 groups in total (Groups 2, 3, 5, 6)
	7. Includes emergency communication expectations in all orientation of nurses to a workplace	Coverage: 0 groups in total; Noncoverage: 24 groups in total
	8. Collaborates with disaster leadership team(s) to develop event‐specific media messages	Coverage: 2 groups in total (Groups 18, 19); Noncoverage: 22 groups in total
	9. Develops guidance on critical documentation to be maintained during disaster or emergency	Coverage: 2 groups in total (Groups 1, 4); Noncoverage: 22 groups in total

Incident Management	10. Participates in development of organizational incident plan consistent with national standards	Coverage: 6 groups in total (Groups 9, 12, 13, 14, 15, 16); Noncoverage: 18 groups in total
	11. Participates with others in post‐event (actual or exercise) evaluation	Coverage: 2 groups in total (Groups 4, 20); Noncoverage: 22 groups in total
	12. Develops action plans for improvement in nursing practice based on event assessment	Coverage: 1 group in total (Group 20); Noncoverage: 23 groups in total
	13. Includes emergency planning guidance when reassigning staff or including unfamiliar colleagues or volunteers	Coverage: 21 groups in total; Noncoverage: 3 groups in total (Groups 3, 6, 21)

Safety and Security	14. Implements materials that support nursing decision‐making that maintains safety during disaster/emergency events	Coverage: 0 groups in total
	15. Provides timely alternative infection control practices applicable within limited resources	Coverage: 0 groups in total
	16. Collaborates with others to facilitate nurses’ access to medical and/or mental health treatment, and other support services as needed	Coverage: 1 group in total (Group 6); Noncoverage: 23 groups in total
	17. Explains the levels/differences in PPE and indications for use to nurses and others	Coverage: 0 groups in total
	18. Creates an action plan to address and correct/eliminate risks to personal or others’ safety and security	Coverage: 22 groups in total; Noncoverage: 2 groups in total (Groups 19, 20)

Assessment	19. Assures that all nurses have up‐to‐date information on potential emergency events and the process for reporting them if observed	Coverage: 24 groups in total
	20. Develops event‐specific guidance on rapid physical and mental health assessment of individual patients/families/communities based on available information	Coverage: 24 groups in total
	21. Includes principles of disaster/emergency triage in all assessment courses taught in basic and continuing education programs	Coverage: 1 group in total (Group 3); Noncoverage: 23 groups in total
	22. Identifies event‐specific vulnerable population(s) and actions needed to protect them	Coverage: 13 groups in total (Groups 1, 6, 9, 11, 12, 17, 18, 19, 20, 21, 22, 23, 24); Noncoverage: 11 groups in total

Intervention	23. Assures that emergency plans and institutional policy include the expectation that basic first aid can be administered by all nurses	Coverage: 16 groups in total; Noncoverage: 8 groups in total (Groups 1, 2, 3, 4, 5, 6, 7, 8)
	24. Includes organizationally specific guidance on implementation of isolation in an emergency	Coverage: 0 groups in total
	25. Describes the range of CBRNE 10 exposures and the exposure‐related decontamination methods to be used	Coverage: 0 groups in total
	26. Plans for expanded patient, patient’s family or volunteer participation in extending resources in emergency/disaster plan	Coverage: 0 groups in total
	27. Guides implementation of nursing reassignments within an organization’s emergency plan	Coverage: 16 groups in total; Noncoverage: 8 groups in total (Groups 2, 3, 4, 5, 6, 7, 8, 21)
	28. Guides nursing participation in surge activities when required by event	Coverage: 10 groups in total (Groups 1, 3, 4, 5, 6, 7, 8, 18, 20, 21); Noncoverage: 14 groups in total

Recovery	29. Communicates nursing roles, responsibilities and needs to leadership throughout the recovery phase	Coverage: 0 groups in total
	30. Maintains up‐to‐date referral resource lists and adds event‐specific modifications as needed	Coverage: 0 groups in total

Law and Ethics	31. Participates in development of emergency‐specific policy and procedure guidance for nurses within the organization/institution	Coverage: 0 groups in total
	32. Participates in the development of disaster/emergency frameworks for allocation of resources (e.g., staff, supplies, medications)	Coverage: 24 groups in total
	33. Develops guidance and support for nurses expected to apply utilitarian principles in practice during emergency and disaster response	Coverage: 24 groups in total

In the Preparation and Planning domain, Items 1 and 3 were covered in all 24 scripts. These scripts described multiagency participation in drill planning and communication of nurses’ roles and responsibilities during the emergency response. Item 4 was covered in 15 scripts. Relevant script content included measures addressing the needs of vulnerable populations, such as prioritizing pregnant women for transfer or providing event‐specific care for infants. Item 2 was covered in one script, and Item 5 was not covered in any script. No script explicitly addressed the incorporation of Level I disaster nursing competencies into basic nursing education or refresher courses.

2. Communication: textual coverage of communication systems, communication training, media messages, and documentation guidance

In the Communication domain, Item 6 was covered in 20 scripts. These scripts described emergency communication tools or processes, such as command platforms, walkie‐talkies, megaphones, fluorescent batons, chip‐embedded wristbands, or other scenario‐specific communication arrangements. Item 7 was not covered in any script. Items 8 and 9 were each covered in two scripts. No script explicitly addressed emergency communication expectations in workplace orientation. Event‐specific media message development and guidance on critical documentation during disasters or emergencies were represented in a small number of scripts.

3. Incident Management: Textual coverage of incident planning, post‐event evaluation, improvement planning, and staff reassignment guidance

In the Incident Management domain, Item 13 was covered in 21 scripts. Representative script excerpts included guidance for reassigned staff, unfamiliar colleagues, or volunteers during the emergency response, such as instructions on monitoring vital signs or applying zone‐specific emergency procedures. Item 10 was covered in six scripts. Item 11 was covered in two scripts, and Item 12 was covered in one script. Most scripts did not explicitly describe participation in post‐event evaluation or the development of nursing practice improvement plans based on event assessment.

4. Safety and Security: textual coverage of decision support, infection control, staff support, PPE guidance, and safety risk control

In the Safety and Security domain, Item 18 was covered in 22 scripts. Examples included actions to identify, correct, or reduce safety risks, such as placing warning signs, using nonslip mats, setting up rain shelters, or monitoring environmental hazards. Items 14, 15, and 17 were not covered in any script. Item 16 was covered in one script. No script explicitly addressed decision‐support materials for nursing safety, alternative infection control practices under limited resources, or guidance explaining the levels and indications for personal protective equipment.

5. Assessment: textual coverage of information reporting, rapid assessment, triage education, and vulnerable population identification

In the Assessment domain, Items 19 and 20 were covered in all 24 scripts. These scripts described processes for receiving and reporting emergency information and included event‐specific guidance for rapid physical or mental health assessment, including triage‐related assessment arrangements. Item 22 was covered in 13 scripts. Relevant script excerpts described the identification of event‐specific vulnerable populations, such as pregnant women and children, and corresponding protective or assessment measures. Item 21 was covered in one script. Explicit reference to incorporating disaster or emergency triage principles into basic or continuing education assessment courses was uncommon in the analyzed scripts.

6. Intervention: textual coverage of first aid, isolation guidance, decontamination, volunteer planning, reassignment, and surge support

In the Intervention domain, Items 23 and 27 were each covered in 16 scripts. The scripts described expectations regarding basic first aid and guidance for nursing reassignment within the emergency plan. Item 28 was covered in 10 scripts and included descriptions of nursing participation in surge or reinforcement activities. Items 24, 25, and 26 were not covered in any script. No script explicitly addressed organizational guidance on emergency isolation, CBRNE exposure‐related decontamination methods, or planned participation of patients, family members, or volunteers to extend resources.

7. Recovery: textual coverage of recovery‐phase nursing roles and referral resource lists

In the Recovery domain, Items 29 and 30 were not covered in any of the 24 scripts. No script explicitly addressed communication of nursing roles, responsibilities, and needs to leadership during the recovery phase. Similarly, referral resource lists and event‐specific modifications were not addressed in any script.

8. Law and Ethics: textual coverage of emergency‐specific policy guidance, resource allocation, and utilitarian principles

In the Law and Ethics domain, Items 32 and 33 were covered in all 24 scripts. The scripts addressed resource allocation based on triage categories, staffing distribution, and prioritization of supplies or procedures according to patient acuity. Item 31 was not covered in any script. No script explicitly addressed the development of emergency‐specific policy and procedure guidance for nurses within the organization or institution.

## 4. Discussion

This study was based on the ICN Core Competencies in Disaster Nursing Version 2.0 framework and involved a textual mapping of drill scripts from 24 teams participating in a single provincial‐level competition. All teams developed their drill scripts based on the same disaster scenario, and the participating institutions were primarily tertiary hospitals. The study assessed the textual coverage of the 8 core competency domains and 33 specific competency items in the drill scripts.

Notably, even in scripts developed by teams with relatively extensive disaster response experience, the textual representation of different disaster nursing core competency domains remained markedly uneven. This study assessed whether disaster nursing core competency‐related content was represented in the drill scripts, rather than whether nurses actually possessed or demonstrated the corresponding competencies; nevertheless, such textual representation may, to some extent, indirectly reflect participants’ understanding and application of relevant disaster nursing competencies. Therefore, the issues identified through textual mapping cannot simply be attributed to deficiencies in individual nurses’ competencies, as they may also have been influenced by factors such as drill scenario design, script development, and the manner in which competency‐related content was represented. Whether these patterns further reflect relationships among hospital emergency management systems, role‐specific competency requirements, and disaster nursing training frameworks requires further investigation.

At the level of textual representation, the drill scripts focused mainly on acute emergency response, with relatively limited attention to continuous improvement, staff support, safety protection, and post‐disaster recovery. However, some competency‐related content, particularly that involving organizational planning, education and training, post‐exercise activities, and recovery‐phase activities, may fall outside the temporal and operational scope of an immediate casualty‐response scenario. Their limited or absent representation should therefore not necessarily be interpreted as deficiencies in emergency preparedness. The Federal Emergency Management Agency (FEMA) disaster management framework emphasizes the use of after action reviews (AARs) and improvement plans to capture lessons from disaster response and support continuous improvement in emergency preparedness [24]. Accordingly, hospital managers could further integrate ICN disaster nursing core competency requirements into emergency plans, role responsibilities, training programs, and evaluation systems. They should also strengthen drill debriefing, corrective actions, continuous quality improvement, and nurses’ involvement in revising emergency plans, optimizing processes, and improving drills.

This study found relatively high textual coverage in the domains of “Assessment,” “Incident Management,” “Preparation and Planning,” and “Communication.” Previous studies based on the ICN disaster nursing core competency framework have similarly shown that nurses tend to place greater emphasis on immediate response capabilities, such as risk identification, communication and coordination, and on‐site management during disasters [25]. “Preparation and Planning” has also been identified as one of the most important core competencies for nurses. Nurses are expected to understand disaster plans and be able to activate emergency plans, communicate and coordinate effectively, and adjust response strategies as circumstances evolve [26].

However, relatively high domain‐level coverage did not mean that all related content was adequately represented. Some content related to organizational planning, training and education, and post‐drill evaluation and improvement was less frequently represented. Because such activities may occur before or after the immediate response phase, or require broader organizational processes, their limited representation may partly reflect the scope of the simulated scenario rather than shortcomings in preparedness. In addition, research on capacity building for WHO Emergency Medical Teams (EMTs) has indicated that the absence of standardized training and core competency frameworks may contribute to variation in training content, teaching approaches, and evaluation criteria, whereas systematic, simulation‐based, and interprofessional training can strengthen disaster preparedness and emergency response among health professionals [27]. Other studies have also emphasized that the value of emergency drills lies not only in strengthening on‐site response but also in promoting organizational learning and system improvement through post‐drill feedback, plan revision, and continuous improvement [28].

Emergency communication and communication system planning were also relatively underrepresented in the scripts. In public health emergencies and disaster settings, nurses and nursing managers undertake important roles in risk communication and interdepartmental coordination. In complex information environments, inadequate information management and crisis communication mechanisms may hinder information flow, public trust, and organizational emergency response [29]. Based on these findings, hospitals and nursing managers could refine drill design by giving greater attention to underrepresented areas such as training, drill feedback, and risk communication, while linking drill evaluation with emergency plan revision and continuous quality improvement to support disaster nursing capacity building and strengthen hospital emergency management.

Within the three domains with moderate textual coverage—“Law and Ethics,” “Intervention,” and “Safety and Security”—the drill scripts placed greater emphasis on acute emergency response and resource allocation, while institutional support, staff support, and some safety‐related content were less frequently represented. This pattern may be related to role allocation, organizational culture, and the specific drill scenario. Disaster response usually involves collaboration among medical rescue teams, health authorities, and other agencies. Medical rescue teams mainly focus on on‐site treatment and emergency response, whereas policy development, supervision, and interdepartmental coordination are more often undertaken by health authorities or other management bodies. Such role differentiation may partly explain the limited representation of nurses’ involvement in emergency policy and procedure development and nursing decision support.

Previous studies have reported that nurses may face unclear role boundaries as well as complex ethical and legal challenges during disasters [30], and ethical decision‐making has also been recognized as an important component of disaster nursing competency [31]. Drill design should therefore clarify nurses’ roles in interdepartmental collaboration and decision support while also incorporating relevant ethical and legal issues. It should also be noted that the crowd crush scenario in this study did not involve chemical, biological, radiological, or other hazardous exposures; therefore, the absence of decontamination procedures should be interpreted in light of the specific scenario.

Volunteer management and nurses’ psychological support were also relatively underrepresented. Volunteer management often requires coordination across legal, governmental, and nongovernmental systems [32], which may explain its limited representation in scripts focused mainly on medical rescue and on‐site response. Similarly, limited attention to nurses’ psychological support may reflect the emphasis on immediate tasks such as casualty treatment and resource allocation. Hospitals and nursing managers could further clarify nurses’ roles in interdepartmental coordination, emergency decision‐making, and ethical management, while giving greater attention to volunteer coordination, psychological support for nursing staff, and safety‐related content in emergency plans, training, and drills.

Compared with the domains discussed above, “Recovery” was the only competency domain with no textual coverage. The drill scripts did not address communication with management regarding nurses’ roles, responsibilities, and needs during the recovery phase, nor did they describe maintaining or updating referral resource lists in response to changing post‐event needs. However, the absence of these items should be interpreted in the context of the exercise design. Because the simulated scenario focused primarily on immediate casualty response, recovery‐phase activities may have fallen outside the intended temporal and operational scope of the drill. Their absence from the scripts may therefore reflect the scope and focus of the simulated scenario rather than inadequate emergency preparedness.

Although recovery‐related activities were outside or less central to the immediate response focus of this exercise, recovery remains an important component of the broader disaster management cycle. Previous reviews have shown that nurses continue to undertake responsibilities after disasters, including infection prevention, psychological support, casualty transfer, shelter management, and rehabilitation [33]. Recovery is therefore an important component of disaster nursing and should be more systematically incorporated into training and emergency management. Hospitals and nursing managers could further clarify recovery‐phase nursing responsibilities in emergency plans, training, and drills, while strengthening post‐disaster needs assessment, psychological support, referral coordination, rehabilitation linkage, resource updating, information feedback, and interorganizational collaboration.

## 5. Limitations

This study has several limitations. First, the data were derived from a regularly held large‐scale provincial disaster emergency drill competition, in which the drill content was designed primarily according to the predominant types of disasters and regional disaster characteristics of the local area at the time. Therefore, the study has a certain degree of regional specificity, which may limit the regional representativeness of the findings. Accordingly, the findings may not fully represent conditions in other regions and should be interpreted with caution when generalized to other healthcare settings or disaster scenarios. It should also be noted that this study analyzed the textual representation of disaster nursing core competency‐related content in drill scripts developed under standardized simulated disaster scenarios, rather than directly measuring participants’ actual performance during drills or real‐world disasters. Although the representation of competency‐related content in the scripts may, to some extent, indirectly reflect participants’ understanding and application of relevant disaster nursing competencies, the absence or limited representation of a competency item in a script should not be interpreted as evidence that nurses or medical rescue teams lack the corresponding competency. This consideration is particularly relevant to competencies involving organizational planning, education and training, post‐exercise activities, and recovery‐phase activities, which may fall outside the temporal and operational scope of an immediate casualty‐response simulation. In addition, differences in script‐writing style, level of detail, and mode of expression across teams may have influenced the textual coverage results. Finally, this study mapped 24 mass‐casualty incident drill scripts against the ICN Core Competencies in Disaster Nursing Version 2.0. Although a standardized framework was used for coding and classification, some subjectivity may have remained in textual interpretation and item assignment. Moreover, the findings were not validated using interviews, direct observation, or actual drill performance data; therefore, the extent to which textual representation in the scripts corresponds to actual disaster nursing competency cannot be determined. Future studies could include a wider range of regions, healthcare institutions at different levels, and disaster scenarios, and incorporate multiple data sources, such as interviews, direct observation, and actual drill evaluations, to further validate the findings.

## 6. Conclusion

Based on the mapping of 24 emergency drill scripts for crowd crush incidents against the ICN Core Competencies in Disaster Nursing Version 2.0, Assessment, Incident Management, Preparation and Planning, and Communication showed relatively high textual coverage, whereas Law and Ethics, Intervention, and Safety and Security showed moderate textual coverage, and Recovery was not represented. Future drill design should strengthen competency‐related content that can reasonably be demonstrated during immediate response but was relatively underrepresented, particularly staff safety and support, communication‐related training and guidance, and ethical and legal decision support. Competencies involving organizational planning, education and training, post‐exercise activities, and recovery‐phase activities may not be fully captured through immediate casualty‐response scripts and should therefore be assessed through complementary approaches, including relevant planning and training activities, post‐exercise evaluation, recovery‐focused exercises, direct observation, and drill‐performance assessment. Such complementary assessment may provide a more objective and comprehensive basis for nursing leadership, disaster preparedness planning, workforce development, competency assessment, and organizational decision‐making.

### 6.1. Implications for Nursing Management Section

This study may help nursing managers understand how disaster nursing core competencies are represented in emergency drill scripts and identify competency areas requiring further attention. The findings may inform targeted improvements in disaster preparedness planning, training, drill design, and subsequent competency assessment. Competencies that are difficult to capture through immediate casualty‐response scripts, particularly those involving organizational planning, education and training, post‐exercise activities, and recovery‐phase activities, may require complementary approaches such as planning and training activities, post‐exercise review, and recovery‐focused exercises.

## Funding

This study received no external funding.

## Conflicts of Interest

The authors declare no conflicts of interest.

## Supporting Information

Additional supporting information can be found online in the Supporting Information section.

## Supporting information


**Supporting Information** The supporting Information include five tables. Table S1 presents the domains and specific competency items of the ICN Core Competencies in Disaster Nursing Version 2.0. Table S2 describes the demonstrability of the 33 ICN Level II competencies within the simulated crowd crush mass‐casualty response scenario. Table S3 provides an example of textual mapping in the Assessment domain. Table S4 presents the mapping of emergency drill script excerpts to the corresponding ICN competency items, while Table S5 provides the original script used for this mapping.

## Data Availability

The data that support the findings of this study are available from the corresponding author upon reasonable request.
