# Supplementary material for: A Textual Mapping Analysis of Mass Trampling Incident Emergency Drill Scripts Based on ICN Core Competencies in Disaster Nursing Version 2.0
Source: J Nurs Manag. 2026 Oct 29;2026:5309874. doi: 10.1155/jonm/5309874 (PMC13720332; doi:10.1155/jonm/5309874)
Supplement: Supplementary file 1 [file JONM-2026-5309874-s001.docx]

Table S1. Domains and Specific Competency Items of the ICN Core Competencies in Disaster Nursing Version 2.0

| **Domain** | **Item** |
| --- | --- |
| Domain 1: Preparation and Planning | 1.1 Participates with other disciplines in planning emergency drills/exercises at the institution or community level at least annually |
|  | 1.2 Plans nursing improvement actions based on results of drill/exercise evaluation |
|  | 1.3 Communicates roles and responsibilities of nurses to others involved in planning, preparation, response and recovery |
|  | 1.4 Includes actions relevant to needs of vulnerable populations in emergency plans |
|  | 1.5 Incorporates Level I core competencies in Disaster Nursing in any basic nursing education programme or refresher course |
| Domain 2: Communication | 2.1 Plans for adaptable emergency/disaster communications systems |
|  | 2.2 Includes emergency communication expectations in all orientation of nurses to a workplace |
|  | 2.3 Collaborates with disaster leadership team(s) to develop event-specific media messages |
|  | 2.4 Develops guidance on critical documentation to be maintained during disaster or emergency |
| Domain 3: Incident Management | 3.1 Participates in development of organisational incident plan consistent with national standards |
|  | 3.2 Participates with others in post-event (actual or exercise) evaluation |
|  | 3.3 Develops action plans for improvement in nursing practice based on event assessment |
|  | 3.4 Includes emergency planning guidance when reassigning staff or including unfamiliar colleagues or volunteers |
| Domain 4: Safety and Security | 4.1 Implements materials that support nursing decision-making that maintains safety during disaster/emergency events |
|  | 4.2 Provides timely alternative infection control practices applicable within limited resources |
|  | 4.3 Collaborates with others to facilitate nurses’ access to medical and/or mental health treatment, and other support services as needed |
|  | 4.4 Explains the levels/differences in PPE and indications for use to nurses and others |
|  | 4.5 Creates an action plan to address and correct/eliminate risks to personal or others’ safety and security |
| Domain 5: Assessment | 5.1 Assures that all nurses have up-to-date information on potential emergency events and the process for reporting them if observed |
|  | 5.2 Develops event-specific guidance on rapid physical and mental health assessment of individual patients/families/communities based on available information |
|  | 5.3 Includes principles of disaster/emergency triage in all assessment courses taught in basic and continuing education programmes |
|  | 5.4 Identifies event-specific vulnerable population(s) and actions needed to protect them |
| Domain 6: Intervention | 6.1 Assures that emergency plans and institutional policy include the expectation that basic first aid can be administered by all nurses |
|  | 6.2 Includes organisationally specific guidance on implementation of isolation in an emergency |
|  | 6.3 Describes the range of CBRNE exposures and the exposure-related decontamination methods to be used |
|  | 6.4 Plans for expanded patient, patient’s family or volunteer participation in extending resources in emergency/disaster plan |
|  | 6.5 Guides implementation of nursing reassignments within an organisation’s emergency plan |
|  | 6.6 Guides nursing participation in surge activities when required by event |
| Domain 7: Recovery | 7.1 Communicates nursing roles, responsibilities and needs to leadership throughout the recovery phase |
|  | 7.2 Maintains up-to-date referral resource lists and adds event-specific modifications as needed |
| Domain 8: Law and Ethics | 8.1 Participates in development of emergency-specific policy and procedure guidance for nurses within the organisation/institution |
|  | 8.2 Participates in the development of disaster/emergency frameworks for allocation of resources (e.g. staff, supplies, medications) |
|  | 8.3 Develops guidance and support for nurses expected to apply utilitarian principles in practice during emergency and disaster response |

Table S2. Demonstrability of the 33 ICN Level II Competencies within the Simulated Crowd Crush Mass-Casualty Response Scenario

| **Domain** | **Item** | **Demonstrability within the Simulated Scenario** |
| --- | --- | --- |
| Domain 1: Preparation and Planning | 1.1 Participates with other disciplines in planning emergency drills/exercises at the institution or community level at least annually | Primarily requires evidence beyond the simulated scenario |
|  | 1.2 Plans nursing improvement actions based on results of drill/exercise evaluation | Post-exercise activity |
|  | 1.3 Communicates roles and responsibilities of nurses to others involved in planning, preparation, response and recovery | Conditionally demonstrable within the scenario |
|  | 1.4 Includes actions relevant to needs of vulnerable populations in emergency plans | Organizational planning |
|  | 1.5 Incorporates Level I core competencies in Disaster Nursing in any basic nursing education programme or refresher course | Education and training activity |
| Domain 2: Communication | 2.1 Plans for adaptable emergency/disaster communications systems | Organizational planning |
|  | 2.2 Includes emergency communication expectations in all orientation of nurses to a workplace | Education and training activity |
|  | 2.3 Collaborates with disaster leadership team(s) to develop event-specific media messages | Conditionally demonstrable within the scenario |
|  | 2.4 Develops guidance on critical documentation to be maintained during disaster or emergency | Organizational planning |
| Domain 3: Incident Management | 3.1 Participates in development of organisational incident plan consistent with national standards | Organizational planning |
|  | 3.2 Participates with others in post-event (actual or exercise) evaluation | Post-exercise activity |
|  | 3.3 Develops action plans for improvement in nursing practice based on event assessment | Post-exercise activity |
|  | 3.4 Includes emergency planning guidance when reassigning staff or including unfamiliar colleagues or volunteers | Directly demonstrable within the scenario |
| Domain 4: Safety and Security | 4.1 Implements materials that support nursing decision-making that maintains safety during disaster/emergency events | Conditionally demonstrable within the scenario |
|  | 4.2 Provides timely alternative infection control practices applicable within limited resources | Directly demonstrable within the scenario |
|  | 4.3 Collaborates with others to facilitate nurses’ access to medical and/or mental health treatment, and other support services as needed | Primarily requires evidence of organizational support |
|  | 4.4 Explains the levels/differences in PPE and indications for use to nurses and others | Directly demonstrable within the scenario |
|  | 4.5 Creates an action plan to address and correct/eliminate risks to personal or others’ safety and security | Conditionally demonstrable within the scenario |
| Domain 5: Assessment | 5.1 Assures that all nurses have up-to-date information on potential emergency events and the process for reporting them if observed | Primarily pertains to the preparedness phase |
|  | 5.2 Develops event-specific guidance on rapid physical and mental health assessment of individual patients/families/communities based on available information | Conditionally demonstrable within the scenario |
|  | 5.3 Includes principles of disaster/emergency triage in all assessment courses taught in basic and continuing education programmes | Education and training activity |
|  | 5.4 Identifies event-specific vulnerable population(s) and actions needed to protect them | Directly demonstrable within the scenario |
| Domain 6: Intervention | 6.1 Assures that emergency plans and institutional policy include the expectation that basic first aid can be administered by all nurses | Organizational policy |
|  | 6.2 Includes organisationally specific guidance on implementation of isolation in an emergency | Organizational planning |
|  | 6.3 Describes the range of CBRNE exposures and the exposure-related decontamination methods to be used | Hazard-specific |
|  | 6.4 Plans for expanded patient, patient’s family or volunteer participation in extending resources in emergency/disaster plan | Conditionally demonstrable / organizational planning |
|  | 6.5 Guides implementation of nursing reassignments within an organisation’s emergency plan | Directly demonstrable within the scenario |
|  | 6.6 Guides nursing participation in surge activities when required by event | Directly demonstrable within the scenario |
| Domain 7: Recovery | 7.1 Communicates nursing roles, responsibilities and needs to leadership throughout the recovery phase | Recovery-phase activity |
|  | 7.2 Maintains up-to-date referral resource lists and adds event-specific modifications as needed | Organizational preparedness / recovery |
| Domain 8: Law and Ethics | 8.1 Participates in development of emergency-specific policy and procedure guidance for nurses within the organisation/institution | Organizational policy |
|  | 8.2 Participates in the development of disaster/emergency frameworks for allocation of resources (e.g. staff, supplies, medications) | Organizational planning |
|  | 8.3 Develops guidance and support for nurses expected to apply utilitarian principles in practice during emergency and disaster response | Directly demonstrable within the scenario, but requires strong supporting evidence |

Note. This study-specific classification indicates whether each ICN Level II competency could be demonstrated within the simulated scenario.

Table S3. Example of textual mapping of drill script excerpts to ICN disaster nursing competency items in the Assessment domain

| **Domain** | **Item** | **Script Excerpts** |
| --- | --- | --- |
| Domain 5: Assessment | 5.1 Assures that all nurses have up-to-date information on potential emergency events and the process for reporting them if observed | Team Leader: Command Center, this is Rescue Team 1. We have arrived at the scene of the stampede incident. Traffic police have implemented traffic control, secured the area, and evacuated bystanders. Some casualties have already made their own way to nearby hospitals for treatment. There are eight casualties currently visible at the scene. Firefighters have moved all eight casualties to a safe area. We are now preparing to immediately conduct triage and provide emergency treatment. We request immediate additional support. Over. (Holding a two-way radio)  Narrator: After completing the emergency assessment of all casualties at the scene, the rescue team members immediately initiated the reporting process. They reported the condition of all casualties to the rescue team leader and requested further instructions regarding emergency treatment and transport.  Team Member No. 3: Reporting to the Captain, the site environment is safe.  Team Member No. 2: Reporting to the Captain, the drone patrol has identified and eliminated any safety hazards; the site is safe.  Team Member 3: Reporting to the Captain: Casualty 6 is exhibiting abnormal breathing, flail chest and a fracture of the left lower limb. Should we immediately perform thoracic decompression, immobilize the lower-limb fracture, and request an ambulance for transfer and further treatment? Please advise.  Team Member 2: Reporting to the Captain, Patient 5 is complaining of neck pain and numbness in the limbs; cervical spine injury is suspected. Should we immediately immobilize the cervical spine and arrange transfer for further treatment? Please advise. |
|  | 5.2 Develops event-specific guidance on rapid physical and mental health assessment of individual patients/families/communities based on available information | Team Leader: Hello everyone! We are 120 emergency medical personnel. Please remain calm, do not panic, and do not move about. We will now begin treating you immediately. Please follow my instructions. Thank you. Now, please follow my first instruction: anyone who can walk unaided, please come to my right-hand side.  Team Leader: Copy that. Team Member No. 1, please take these four casualties to the green zone for assessment.  Team Leader: To the remaining casualties, please remain calm and listen for my next instructions. Anyone who can hear me, please wave your hands and shout for help. Team members 2, 3 and 4, please assess casualties 5, 6 and 7 respectively.  Narrator: The team leader issues instructions based on a brief triage assessment to evaluate the condition of each casualty. |
|  | 5.3 Includes principles of disaster/emergency triage in all assessment courses taught in basic and continuing education programmes | / |
|  | 5.4 Identifies event-specific vulnerable population(s) and actions needed to protect them | / |

Table S4. Mapping of Emergency Drill Script Excerpts to the ICN Core Competencies in Disaster Nursing, Version 2.0

| **Domain** | **Item** | **Script Excerpts** |
| --- | --- | --- |
| Domain 1: Preparation and Planning | 1.1 Participates with other disciplines in planning emergency drills/exercises at the institution or community level at least annually | Narrator: To strengthen emergency preparedness and response capacity, our institution annually organises or participates in emergency drills in response to major local disaster events. These drills are conducted in collaboration with government agencies, professional medical associations, healthcare institutions, community organisations, and other relevant professional organisations. Nursing personnel work collaboratively with other relevant professionals in planning and developing the drill programme. For example, in 2025, taking into account the emergency preparedness requirements associated with China’s 15th National Games and the series of disasters caused by the high incidence of heavy rainfall in Guangdong Province, China, during July, the participating professionals jointly determined the scenario for this exercise: a mass-casualty crowd crush incident triggered by sudden heavy rainfall as spectators were leaving a sporting event at a stadium.  PPT: At 16:40 on 16 July 2025, a sudden downpour struck as spectators were leaving a sports meet at a stadium. This triggered a stampede, trapping approximately 50 people in a crowded area. Following the incident, you arrived at the scene as part of the first rescue team and carried out rescue operations in an orderly manner. |
|  | 1.2 Plans nursing improvement actions based on results of drill/exercise evaluation | / |
|  | 1.3 Communicates roles and responsibilities of nurses to others involved in planning, preparation, response and recovery | Team Leader: Hello everyone! We are 120 emergency medical personnel. Please remain calm, do not panic, and do not move about. We will now begin treating you immediately. Please follow my instructions. Thank you. Now, please follow my first instruction: anyone who can walk unaided, please come to my right-hand side.  Team Leader: Copy that. Team Member No. 1, please take these four casualties to the green zone for assessment.  Team Leader: To the remaining casualties, please remain calm and listen for my next instructions. Anyone who can hear me, please wave your hands and shout for help. Team members 2, 3 and 4, please assess casualties 5, 6 and 7 respectively. |
|  | 1.4 Includes actions relevant to needs of vulnerable populations in emergency plans | / |
|  | 1.5 Incorporates Level I core competencies in Disaster Nursing in any basic nursing education programme or refresher course | / |
| Domain 2: Communication | 2.1 Plans for adaptable emergency/disaster communications systems | Team Leader: Reporting to the command centre: Rescue Team 1 has arrived at the scene of the stampede. Traffic police have implemented traffic control measures and cordoned off the area. The crowd has been evacuated. Some of the injured have made their own way to nearby hospitals. There are eight injured persons at the scene, as observed. The fire service has moved all eight to a safe area. Requesting backup. Over and out. (Handheld radio) |
|  | 2.2 Includes emergency communication expectations in all orientation of nurses to a workplace | / |
|  | 2.3 Collaborates with disaster leadership team(s) to develop event-specific media messages | / |
|  | 2.4 Develops guidance on critical documentation to be maintained during disaster or emergency | / |
| Domain 3: Incident Management | 3.1 Participates in development of organisational incident plan consistent with national standards | / |
|  | 3.2 Participates with others in post-event (actual or exercise) evaluation | / |
|  | 3.3 Develops action plans for improvement in nursing practice based on event assessment | / |
|  | 3.4 Includes emergency planning guidance when reassigning staff or including unfamiliar colleagues or volunteers | Team Member 1: Reporting to the Team Leader, treatment of the casualty in the green zone is complete. Please advise.  Team Leader: Copy. Please assist with Patient 5.  Team Leader: Copy that. Assist Patient 6 and prepare the spinal board.  Team Leader: Copy that. Team Members 2 and 3, take the deceased patient to the black zone.  Team Leader: Team members 1 and 4, reassess the condition of the critically injured patient every five minutes. |
| Domain 4: Safety and Security | 4.1 Implements materials that support nursing decision-making that maintains safety during disaster/emergency events | / |
|  | 4.2 Provides timely alternative infection control practices applicable within limited resources | / |
|  | 4.3 Collaborates with others to facilitate nurses’ access to medical and/or mental health treatment, and other support services as needed | / |
|  | 4.4 Explains the levels/differences in PPE and indications for use to nurses and others | / |
|  | 4.5 Creates an action plan to address and correct/eliminate risks to personal or others’ safety and security | Team Leader: Assemble immediately. We have received notification from the 120 Command Centre to proceed straight to the stampede site. On my command: march, halt, about turn, align to the left, face forward, roll call: 1, 2, 3, 4, 5. Rescue Team 1: 5 members expected, 5 present. Assembly complete. The time is 16:48. Safety officer, please assess the site conditions.  Narrator: One safety officer is conducting a safety assessment of the ground conditions, whilst another is using a drone to inspect the scene for safety hazards.  Team Member No. 3: Reporting to the Captain, the site environment is safe.  Team Member No. 2: Reporting to the Captain, the drone patrol has identified and eliminated any safety hazards; the site is safe. |
| Domain 5: Assessment | 5.1 Assures that all nurses have up-to-date information on potential emergency events and the process for reporting them if observed | Team Leader: Command Center, this is Rescue Team 1. We have arrived at the scene of the stampede incident. Traffic police have implemented traffic control, secured the area, and evacuated bystanders. Some casualties have already made their own way to nearby hospitals for treatment. There are eight casualties currently visible at the scene. Firefighters have moved all eight casualties to a safe area. We are now preparing to immediately conduct triage and provide emergency treatment. We request immediate additional support. Over. (Holding a two-way radio)  Narrator: After completing the emergency assessment of all casualties at the scene, the rescue team members immediately initiated the reporting process. They reported the condition of all casualties to the rescue team leader and requested further instructions regarding emergency treatment and transport.  Team Member No. 3: Reporting to the Captain, the site environment is safe.  Team Member No. 2: Reporting to the Captain, the drone patrol has identified and eliminated any safety hazards; the site is safe.  Team Member 3: Reporting to the Captain: Casualty 6 is exhibiting abnormal breathing, flail chest and a fracture of the left lower limb. Should we immediately perform thoracic decompression, immobilize the lower-limb fracture, and request an ambulance for transfer and further treatment? Please advise.  Team Member 2: Reporting to the Captain, Patient 5 is complaining of neck pain and numbness in the limbs; cervical spine injury is suspected. Should we immediately immobilize the cervical spine and arrange transfer for further treatment? Please advise. |
|  | 5.2 Develops event-specific guidance on rapid physical and mental health assessment of individual patients/families/communities based on available information | Team Leader: Hello everyone! We are 120 emergency medical personnel. Please remain calm, do not panic, and do not move about. We will now begin treating you immediately. Please follow my instructions. Thank you. Now, please follow my first instruction: anyone who can walk unaided, please come to my right-hand side.  Team Leader: Copy that. Team Member No. 1, please take these four casualties to the green zone for assessment.  Team Leader: To the remaining casualties, please remain calm and listen for my next instructions. Anyone who can hear me, please wave your hands and shout for help. Team members 2, 3 and 4, please assess casualties 5, 6 and 7 respectively.  Narrator: The team leader issues instructions based on a brief triage assessment to evaluate the condition of each casualty. |
|  | 5.3 Includes principles of disaster/emergency triage in all assessment courses taught in basic and continuing education programmes | / |
|  | 5.4 Identifies event-specific vulnerable population(s) and actions needed to protect them | / |
| Domain 6: Intervention | 6.1 Assures that emergency plans and institutional policy include the expectation that basic first aid can be administered by all nurses | Team Member 3: Reporting to the Captain: Casualty 6 is exhibiting abnormal breathing, flail chest and a fracture of the left lower limb. Please advise.  Team Leader: Copy that. Perform a thoracentesis to release air immediately and secure the airway.  Team Member 2: Reporting to the Captain, Patient 5 is complaining of neck pain and numbness in the limbs; cervical spine injury is suspected. Please advise.  Team Leader: Copy! Treat as a cervical injury.  Team Member 3: Thoracentesis complete.  Team Leader: Secure the rib splint immediately.  Team Leader: Rib splinting complete. The patient’s breathing has not improved; prepare for a cricothyroidotomy.  Team Leader: Cricoid laryngotomy completed. Report vital signs.  Team Leader: Prepare for a bone marrow aspiration. |
|  | 6.2 Includes organisationally specific guidance on implementation of isolation in an emergency | / |
|  | 6.3 Describes the range of CBRNE exposures and the exposure-related decontamination methods to be used | / |
|  | 6.4 Plans for expanded patient, patient’s family or volunteer participation in extending resources in emergency/disaster plan | / |
|  | 6.5 Guides implementation of nursing reassignments within an organisation’s emergency plan | Team Member 1: Reporting to the Team Leader, treatment of the casualty in the green zone is complete. Please advise.  Team Leader: Copy. Please assist with Patient 5.  Team Leader: Copy that. Assist Patient 6 and prepare the spinal board. |
|  | 6.6 Guides nursing participation in surge activities when required by event | Team Leader: Assemble immediately. We have received notification from the 120 Command Centre to proceed straight to the stampede site. On my command: march, halt, about turn, align to the left, face forward, roll call: 1, 2, 3, 4, 5. Rescue Team 1: 5 members expected, 5 present. Assembly complete. The time is 16:48. Safety officer, please assess the site conditions. |
| Domain 7: Recovery | 7.1 Communicates nursing roles, responsibilities and needs to leadership throughout the recovery phase | / |
|  | 7.2 Maintains up-to-date referral resource lists and adds event-specific modifications as needed | / |
| Domain 8: Law and Ethics | 8.1 Participates in development of emergency-specific policy and procedure guidance for nurses within the organisation/institution | / |
|  | 8.2 Participates in the development of disaster/emergency frameworks for allocation of resources (e.g. staff, supplies, medications) | Team Leader: Copy that. Team Member No. 1, please take these four casualties to the green zone for assessment.  Team Leader: To the remaining casualties, please remain calm and listen for my next instructions. Anyone who can hear me, please wave your hands and shout for help. Team members 2, 3 and 4, please assess casualties 5, 6 and 7 respectively.  Team Leader: Copy. Please assist with Patient 5.  Team Leader: Copy that. Assist Patient 6 and prepare the spinal board.  Team Member 3: Thoracentesis complete.  Team Leader: Secure the rib splint immediately.  Team Member 3: Copy that!  Team Member No. 3: Reporting to the Captain, Patient No. 6 has been stabilised. Awaiting instructions.  Team Leader: Copy that. Transfer him to Ambulance No. 1 for urgent transport to hospital.  Team Leader: Copy that. Team Members 2 and 3, take the deceased patient to the black zone.  Team Leader: Team members 1 and 4, reassess the condition of the critically injured patient every five minutes. |
|  | 8.3 Develops guidance and support for nurses expected to apply utilitarian principles in practice during emergency and disaster response | Narrator: Command Centre received. Please organise treatment according to the casualties’ conditions and in accordance with the START (Simple Triage and Rapid Treatment) triage principles. Particular attention should be given to vulnerable patients, including pregnant women, children, older adults, and unconscious casualties. Rescue Team 2 is being dispatched to provide on-site support. Please maintain contact. Over. |

Table S5. Demonstration of Rescue Techniques for Mass-Stampede Incidents (Script)

**Demonstration of Rescue Techniques for Mass Stampede Incidents**

**(Script)**

**Date: 16 July 2025**

**Demonstration of Rescue Techniques for Mass Stampede Incidents**

**Drill Theme:** Mass Stampede Rescue Skills Demonstration (12 mins)

**Drill Content:** Triage, trauma first aid (haemostasis and dressing, immobilisation and transport, circulation, airway management), Basic Life Support (BLS), crush injury first aid

**Participants:** Disaster Emergency Rescue Team

**Date of Exercise:** 16 July 2025

**Roles:** Team Leader: On-site Commander (oversees rescue procedures, issues instructions, performs immobilisation, cricothyroidotomy, and bone marrow aspiration)

1. Member No. 2: Haemostasis and bandaging, immobilisation and transport
2. Member No. : Safety Officer, Patient Handling, Immobilisation and Transport
3. Member No. : Thoracentesis, immobilisation and transport, cricothyroid membrane incision, bone marrow aspiration
4. Team Member No. : Haemostasis, dressing, immobilisation and transport

**Total number of casualties: 8**

| **Zone** | **Casualty ID** | **Simulated Injury** | **Skills Demonstrated** |
| --- | --- | --- | --- |
| Green Zone | 1 | Forearm abrasion | Bandage, bandage |
|  | 2 | Knee abrasion | Disinfect, bandage |
|  | 3 | Scalp abrasion, no active bleeding | Disinfection, dressing |
|  | 4 | Abrasion to the right upper limb, with slight oozing | Disinfection, dressing |
| Red zone | 5  6 | Neck injury  Neck injury, tension pneumothorax + shock | Cervical collar immobilisation  Cervical collar immobilisation, large-bore needle decompression, cricothyroidotomy, intramedullary infusion technique |
| Yellow Zone | 7 | Fracture of the left upper limb | Inflatable splint immobilisation, sling immobilisation |
| Black Zone | 8 | Confirmed death | No intervention + evacuation route |

**Narrator:** To strengthen emergency preparedness and response capacity, our institution annually organises or participates in emergency drills in response to major local disaster events. These drills are conducted in collaboration with government agencies, professional medical associations, healthcare institutions, community organisations, and other relevant professional organisations. Nursing personnel work collaboratively with other relevant professionals in planning and developing the drill programme. For example, in 2025, taking into account the emergency preparedness requirements associated with China’s 15th National Games and the series of disasters caused by the high incidence of heavy rainfall in Guangdong Province, China, during July, the participating professionals jointly determined the scenario for this exercise: a mass-casualty crowd crush incident triggered by sudden heavy rainfall as spectators were leaving a sporting event at a stadium.

PPT: At 16:40 on 16 July 2025, a sudden downpour struck as spectators were leaving a sports meet at a stadium. This triggered a stampede, trapping approximately 50 people in a crowded area. Following the incident, you arrived at the scene as part of the first rescue team and carried out rescue operations in an orderly manner.

**Team Leader:** Assemble immediately. We have received notification from the 120 Command Centre to proceed straight to the stampede site. On my command: march, halt, about turn, align to the left, face forward, roll call: 1, 2, 3, 4, 5. Rescue Team 1: 5 members expected, 5 present. Assembly complete. The time is 16:48. Safety officer, please assess the site conditions.

**Team Members 2 and 3:** Copy that!

**Narrator:** One safety officer is conducting a safety assessment of the ground conditions, whilst another is using a drone to inspect the scene for safety hazards.

**Team Member No. 3:** Reporting to the Captain, the site environment is safe.

**Team Leader:** Copy that! Fall in!

Team Member No. 2: Reporting to the Captain, the drone patrol has identified and eliminated any safety hazards; the site is safe.

**Team Leader:** Copy that! Fall in!

**Team Leader:** Command Center, this is Rescue Team 1. We have arrived at the scene of the stampede incident. Traffic police have implemented traffic control, secured the area, and evacuated bystanders. Some casualties have already made their own way to nearby hospitals for treatment. There are eight casualties currently visible at the scene. Firefighters have moved all eight casualties to a safe area. We are now preparing to immediately conduct triage and provide emergency treatment. We request immediate additional support. Over. (Holding a two-way radio)

**Narrator:** Command Centre received. Please organise treatment according to the casualties’ conditions and in accordance with the START (Simple Triage and Rapid Treatment) triage principles. Particular attention should be given to vulnerable patients, including pregnant women, children, older adults, and unconscious casualties. Rescue Team 2 is being dispatched to provide on-site support. Please maintain contact. Over.

**Team Leader:** Copy that. There is no risk of secondary disasters at the scene. We have taken personal protective measures. All team members, please establish the red, yellow, green and black zones respectively, and set up thermal tents.

**Team Members 1, 2, 3 and 4:** Copy that!

**Team Leader:** Hello everyone! We are 120 emergency medical personnel. Please remain calm, do not panic, and do not move about. We will now begin treating you immediately. Please follow my instructions. Thank you. Now, please follow my first instruction: anyone who can walk unaided, please come to my right-hand side.

**Team Member No. 1:** 1, 2, 3, 4. Reporting to the Captain: there are four casualties in total.

**Team Leader:** Copy that. Team Member No. 1, please take these four casualties to the green zone for assessment.

**Team Member No. 1:** Copy that!

**Team Leader:** To the remaining casualties, please remain calm and listen for my next instructions. Anyone who can hear me, please wave your hands and shout for help. Team members 2, 3 and 4, please assess casualties 5, 6 and 7 respectively.

**Team Members 2, 3 and 4:** Copy that!

**Narrator:** The team leader issues instructions based on a brief triage assessment to evaluate the condition of each casualty.

**Team Member 3:** Reporting to the Captain: Casualty 6 is exhibiting abnormal breathing, flail chest and a fracture of the left lower limb. Should we immediately perform thoracic decompression, immobilize the lower-limb fracture, and request an ambulance for transfer and further treatment? Please advise.

**Team Leader:** Copy that. Proceed with immediate thoracic decompression, immobilize the lower-limb fracture, maintain the airway, and arrange ambulance transfer for further treatment.

**Team Member 3:** Copy that!

**Team Member 2:** Reporting to the Captain, Patient 5 is complaining of neck pain and numbness in the limbs; cervical spine injury is suspected. Should we immediately immobilize the cervical spine and arrange transfer for further treatment? Please advise.

**Team Leader:** Copy! Proceed with cervical spine immobilization and arrange transfer for further treatment.

**Team Member 2:** Copy that!

**Team Member 1:** Reporting to the Team Leader, treatment of the casualty in the green zone is complete. Please advise.

**Team Leader:** Copy. Please assist with Patient 5.

**Team Member 1:** Copy.

**Narrator:** After completing the emergency assessment of all casualties at the scene, the rescue team members immediately initiated the reporting process. They reported the condition of all casualties to the rescue team leader and requested further instructions regarding emergency treatment and transport.

**Narrator:** Following triage, the team members are carrying out emergency rescue operations in an orderly manner.

Team Members 1 and 2 are treating Patient 5 for a neck injury,

Team Member 4 is immobilising the fracture in Patient 7’s left upper limb.

Team Member 3 is performing an emergency thoracocentesis to release air from the patient’s chest.

Team Member 4: Reporting to the team leader, Patient 7 has been treated and transferred to the yellow zone thermal tent.

**Team Leader:** Copy that. Assist Patient 6 and prepare the spinal board.

**Team Member 3:** Thoracentesis complete.

**Team Leader:** Secure the rib splint immediately.

**Team Member 3:** Copy that!

**Narrator:** Following emergency thoracentesis to relieve pressure, the patient is still exhibiting paradoxical breathing. The team leader is currently applying a rib splint to stabilise the patient and alleviate the paradoxical breathing. Team Members 1, 2 and 4 are systematically treating the patient with cervical spine injury, applying a cervical collar to minimise the risk of secondary injury.

**Team Leader:** Rib splinting complete. The patient’s breathing has not improved; prepare for a cricothyroidotomy.

**Narrator:** Following the application of the rib splint, Patient 6 was reassessed; symptoms of respiratory distress had not subsided. At this point, the Team Leader urgently performed a cricothyroidotomy on the patient.

**Team Leader:** Cricoid laryngotomy completed. Report vital signs.

**Team Member No. 3:** Copy that. The patient’s oxygen saturation has improved slightly, but blood pressure remains shock-level!

**Team Leader:** Prepare for a bone marrow aspiration.

**Team Member No. 3:** Copy that!

**Narrator:** At this point, Patient 6 suddenly experienced a drop in blood pressure. The team leader urgently performed a bone marrow cavity puncture to establish a non-collapsible airway for the patient.

**Narrator:** Following emergency treatment, Patient 5 with cervical spine injury has been transferred to the Red Zone. Team Members 1 and 2 are assisting with Patient 6, applying a mouldable splint to the left lower limb, whilst Team Member 4 is conducting a dynamic assessment of the condition of patients in all zones.

**Team Member No. 3:** Reporting to the Captain, Patient No. 6 has been stabilised. Awaiting instructions.

**Team Leader:** Copy that. Transfer him to Ambulance No. 1 for urgent transport to hospital.

**Team Member No. 3:** Copy that. On my command: 1, 2, 3, lift; 1, 2, 3, lower. Report to the Captain: the patient has been transferred to Ambulance No. 1. Awaiting further instructions.

**Team Leader:** Copy that. Team Members 2 and 3, take the deceased patient to the black zone.

**Team Members 2 and 3:** Roger that!

**Team Leader:** Team members 1 and 4, reassess the condition of the critically injured patient every five minutes.

**Team Members 1 and 4:** Copy!

**Team Leader:** Reporting to the command centre. It is now 18:37. Rescue Team 1 has completed triage on site. There are 2 critically injured patients, 1 seriously injured, 4 with minor injuries, and 1 deceased. All casualties have been properly attended to. Awaiting instructions.

**Narrator:** Control Centre here. Immediately transfer the critically injured to the nearest hospital for emergency treatment. We have notified the nearest hospital to prepare for their arrival. The remaining casualties are to be handled by Rescue Team 2. Over and out.

**Team Leader:** Copy that. Fall in immediately. Align to the left. Face forward. Roll call: 1, 2, 3, 4, 5. Rescue Team 1: 5 members expected, 5 present. Roll call complete. Rescue operation concluded. (Bows) Turn left. March (exits stage).

**Narrator:** Patients in the Red Zone, Yellow Zone and Green Zone are being transferred to the relevant hospitals in sequence, whilst those in the Black Zone are being handed over to the relevant authorities. Thanks to the prompt activation of the emergency medical response system, all casualties have received effective treatment. The rescue team maintained close contact with the command centre, and on-site organisation and command were orderly, ensuring the situation was effectively brought under control. The treatment of the casualties’ injuries was effective, and their emotions were also successfully managed and stabilised.

**To the judges, the demonstration is now complete. Thank you.**
